# A Monte Carlo method to estimate the confidence intervals for the concentration index using aggregated population register data

**DOI:** 10.1007/s10742-015-0137-1

**Published:** 2015-02-18

**Authors:** Sonja Lumme, Reijo Sund, Alastair H. Leyland, Ilmo Keskimäki

**Affiliations:** 1 The Social and Health Systems Research Unit, The Department of Health and Social Care Systems, The National Institute for Health and Welfare (THL), P.O. Box 30, 00271 Helsinki, Finland; 2 Department of Social Research, Faculty of Social Sciences, Centre for Quantitative Methods, University of Helsinki, P.O. Box 33, 00014 Helsinki, Finland; 3 MRC/CSO Social and Public Health Sciences Unit, University of Glasgow, 200 Renfield Street, Glasgow, G2 3QB Scotland, UK; 4 School of Health Sciences, University of Tampere, Tampere, 33014 Finland

**Keywords:** Monte Carlo simulation, Health and health care register data, Equality, Concentration index, Confidence interval, Amenable mortality

## Abstract

In this paper, we introduce several statistical methods to evaluate the uncertainty in the concentration index (*C*) for measuring socioeconomic equality in health and health care using aggregated total population register data. The *C* is a widely used index when measuring socioeconomic inequality, but previous studies have mainly focused on developing statistical inference for sampled data from population surveys. While data from large population-based or national registers provide complete coverage, registration comprises several sources of error. We simulate confidence intervals for the *C* with different Monte Carlo approaches, which take into account the nature of the population data. As an empirical example, we have an extensive dataset from the Finnish cause-of-death register on mortality amenable to health care interventions between 1996 and 2008. Amenable mortality has been often used as a tool to capture the effectiveness of health care. Thus, inequality in amenable mortality provides evidence on weaknesses in health care performance between socioeconomic groups. Our study shows using several approaches with different parametric assumptions that previously introduced methods to estimate the uncertainty of the *C* for sampled data are too conservative for aggregated population register data. Consequently, we recommend that inequality indices based on the register data should be presented together with an approximation of the uncertainty and suggest using a simulation approach we propose. The approach can also be adapted to other measures of equality in health.

## Background

A major health policy goal in many countries is to reduce disparities in health and in access to and the quality of health care. Measuring these disparities is a challenge. In order to obtain extensive and precise knowledge of inequalities, comprehensive methods to study equality are necessary. Most studies of equality in health and health care, and most methodological papers, have focused on survey data. Register-based data provide another possible source of data for equality studies. So far, good-quality individual-level administrative data including information on socioeconomic status have been available only in a few countries, such as the Nordic countries, but the importance of such data is likely to increase as better information systems increasingly become available and changes in data privacy regulations will enable broader utilization of the individual-level data in other countries. Register-based data are typically secondary data, i.e., they have not been collected for the purposes of specific studies (Sund 2003). Another, possibly even more important, difference is that register-based data often contain total populations instead of samples. In other words, it may be invalid to use statistical methods that assume sampling variation is the source of uncertainty when measuring the phenomenon of interest. For example, when estimating the uncertainty of the measured indicator from sample data, only sampling error is traditionally taken into account. Other possible sources of uncertainty are ignored. When using total population data, such sampling error does not exist. It is, however, obvious that other sources of error exist, since many events such as deaths are assumed to be stochastic and consequently produce a natural variability in vital statistics (Brillinger 1986). In addition, people in the register at one particular time could be seen as a sample of a super-population, and recorded events on these people can be considered to be one of a series of possible results that could have occurred under the same circumstances (Curtin and Klein 1995).

It is, however, a complicated task to assess the uncertainty in the indicator of interest using population-based data (Sørensen et al. 1996). There are multiple sources of errors that can affect the uncertainty, and the sources evidently vary between situations (Sund 2003). The quality of the data is the main influence on uncertainty. Errors in the data may have originated at the stage of registration due to varying practices and accuracy in the processes. Merging different databases, data handling (for example through aggregation of the data), or processing errors may form challenges. Deterioration of the data quality is possible also later in the analysis phase as a result of mistakes in variable coding or programming errors. In addition to data quality, other sources may introduce uncertainty into the indicator such as the definition of the variables or coding practices. What if the variable is recorded correctly, but does not describe the phenomenon under examination for all individuals properly? The uncertainty is commonly quantified using a confidence interval which provides a means of assessing and reporting the uncertainty and is intuitively straightforward to interpret.

The concentration index (*C*) is a widely used indicator for the quantification of socioeconomic equality in health and in the use of health care (e.g., van Doorslaer et al. 1997; Wagstaff 2000; Vikum et al. 2012). The *C* gives comprehensive summary information about the whole distribution of the studied outcome in a single value, which is a particular advantage when making comparisons in time or between genders, areas, countries, or hospitals. In addition, it has the benefit that the level of the inequality can be visualized with the concentration curve.

This paper is about the methodology of the concentration index when measuring socioeconomic equality in health and health care using aggregated register data. The measured health or health care variable can be, for example, deaths, hospitalizations, or certain procedures. In this context, the term “aggregated data” is taken to mean data that are originally individual level and are later grouped by income. Assessment of the register-based estimates involves the above-mentioned uncertainties. Thus, we introduce several techniques to evaluate uncertainty by calculating confidence intervals for the *C* using empirical data, as the majority of the previous studies using and developing the methodology of the *C* have focused on survey data or hypothetical data, which require different methods (for example, see Kakwani et al. 1997; Waters 2000; Burström et al. 2005; van Ourti 2004; Wagstaff 2005; van Doorslaer et al. 2006; Chen and Roy 2009; Clarke and van Ourti 2010; Konings et al. 2010; Chen et al. 2012). In many of these papers, the uncertainty in the *C* has not been assessed, but Kakwani et al. (1997), van Ourti (2004), Chen and Roy (2009), Konings et al. (2010), and Chen et al. (2012) used improved methods to estimate the uncertainty. The uncertainty in the indicator is an essential question, particularly when making comparisons of equality. Our approaches to estimate the uncertainty in the *C* are based on several Monte Carlo simulations. Simulation has previously been shown to be an effective method for the *C* as well as other inequality indices using survey data (Chen et al. 2012; Mills and Zandvakili 1997; Sergeant and Firth 2006; Modarres and Gastwirth 2006; van Ourti and Clarke 2011). We demonstrate the results of this study empirically using an extensive Finnish aggregated register dataset on amenable mortality. We compare our results to the commonly used standard regression method and the improved method developed by Kakwani et al. (1997).

## Methods

The concentration index (*C*) can be used to measure the degree of socioeconomic inequality in health and health care across the distribution of the whole study population (Wagstaff et al. 1989). The index is based on the Gini coefficient, which is used to assess inequality in income or wealth. The *C* is based on the concentration curve *L*(s), which is a tool to visualize the degree of inequality. When using aggregated data, *L*(s) plots the cumulative proportion of the health outcome variable against the cumulative proportion of the population (s), ranked by socioeconomic group (SEG) from the least to the most advantaged. The *C* is defined as twice the area between the diagonal and *L*(s). In a case of complete equality, the *C* gets a value of 0. Negative values indicate a disproportionate concentration of the health outcome among those classed as disadvantaged and vice versa. The *C* is restricted to values between −1 and 1 when the health variable is not binary (Wagstaff 2005).

For aggregated data—in which the groups comprise SEGs and the socioeconomic indicator is measured on an ordinal scale—the quantitative measure of inequality can be estimated as

1 $$C = \frac{2}{y}\sum\limits_{g = 1}^{G} {y_{g} f_{g} R_{g} - 1}$$

where *y*_*g*_ is the health outcome (such as the annual mortality rate) of the *g*th SEG, and *y* is the mean of the *y*_*g*_ across SEGs weighted by the population share *f*_*g*_. The *R*_*g*_ is the relative rank of the SEG, defined as *R*_*g*_ = ∑ _*γ*=1_^*g*−1^ *f*_*γ*_ + 0.5 *f*_*g*_ and indicates the cumulative proportion of the population up to the midpoint of each group interval. In this study, *y*_*g*_ denotes the directly age-standardized amenable mortality rate (per 100,000 person-years) of the *g*th income group: $y_{g} = \sum\nolimits_{i = 1}^{I} {\frac{{d_{ig} }}{{p_{ig} }}} w_{i} ,$ where *i* is the age group, *d*_*ig*_ is the number of deaths and *p*_*ig*_ is the population size in the *i*th age group of the *g*th SEG, *w*_*i*_ is the weight of the age group according to the standard population. The sum of the standard population is 100,000, i.e., ∑ _*i*=1_^*I*^ *w*_*i*_ = 100,000.

The *C* has also been estimated using a weighted least squares method (WLS) (Lerman and Yitzhaki 1984; Wagstaff et al. 1991). The use of aggregated data necessitates the use of weights. The slope parameter *β*_1_ of the regression model has a computational equivalence with the *C* and is obtained from the WLS model

2 $$2\sigma_{R}^{2} \frac{{y_{g} }}{y}\sqrt {p_{g} } = \beta_{0} \sqrt {p_{g} } + \beta_{1} R_{g} \sqrt {p_{g} } + e_{g} ,$$

where *p*_*g*_ is the population size in the *g*th SEG. The size of the weight indicates the power of the information contained in the associated observation. Thus, if SEGs are of equal size, the weights do not have any effect since each group has equal influence on the final estimate. The variance *σ*_*R*_^2^ is the weighted variance of the rank *R*_*g*_, defined as *σ*_*R*_^2^ = ∑ _*g*=1_^*G*^ *f*_*g*_(*R*_*g*_ − 0.5)^2^. This convenient regression method gives the *C*, irrespective of whether the principal model assumptions apply, because the regression method is an artificial technique to calculate the *C* (Kakwani et al. 1997). Thus, the possible serial correlation resulting from the ranked nature of the independent variable (rank is ordered and cumulative) does not affect the estimated regression coefficient. In addition, it is important to note that a linear relationship between the dependent variable and the rank is not necessary due to the artificial nature of this estimation. Both models (1) and (2) can be applied to population or sample data to calculate the *C*.

The standard error of the regression slope in the WLS model (2) describes the variability of the estimate around the unknown slope parameter *β*_1_. However, in order to construct a confidence interval for the *β*_1_ using formula (2), the key WLS regression assumptions should not be violated. The independence of errors may be violated due to the above-mentioned serial correlation causing either under- or overestimated standard errors. The error terms, e.g., in the regression, either have to be normally distributed and independent, or the number of observations in the regression has to be sufficiently large. Usually, when studying equality, the number of SEGs is relatively low (5–20); thus, the latter assumption is unlikely to be met. Due to its simplicity, using this regression method in statistical packages appears to be a conventional means of obtaining confidence intervals also for the *C* (denoted as REG in this study). Kakwani et al. (1997) developed estimators of the standard error of the *C,* which take into account the serial correlation in the data applicable to data drawn from a sample. We denote this technique as KWV in this study.

In this study, we introduce five different Monte Carlo simulation techniques to estimate the confidence interval for the *C* using income as a socioeconomic indicator. These simulation techniques differ from each other in distributional assumptions and in the phase of the simulation process; one technique simulates the outcome variable of the regression method (2), three of them simulate observed events (*d*_*ig*_), and one simulates observed age-adjusted rates (*y*_*g*_). As one of the techniques applies the regression method (2), the rest of the simulation techniques apply either the regression or the formula method (1).

Our techniques can be applied to the datasets where socioeconomic variable is grouped by proportions, for example income quintiles. The SEGs must be defined by the proportions of the person years (ordered by income). The number of person years in each income group can be assumed to be rather stable when using register data due to large datasets. Due to fixed proportions, the ranking is fixed in all our simulation techniques when estimating the uncertainty (the possible miscoding of the income record and health variable) and this allows modeling variation only in the health outcome variable. Consequently, even though the proportions in each income group are fixed, the uncertainty involved in recording income information is incorporated in our method.

The biasing effect of correlation between the rank and the outcome variable is avoided in all our approaches because the standard error is not estimated from the regression model; we simulate the original data to estimate the uncertainty of the *C*. In addition, the advantage of our approaches is that they aim to model the assumed error of the data and the concentration curve and not the error of the slope parameter of the fitted regression line.

One of these simulation techniques was developed in our recent study (Lumme et al. 2012) in which we made a simple assumption of uncertainty around the dependent variable 2*σ*_*R*_^2^(*y*_*g*_/*y*_*g*_ *y*) in Eq. (2). We denote this technique as MC in this study, and it can be applied only to the regression method (2) to estimate the *C*. We accounted for that uncertainty by assuming $2\sigma_{R}^{2} \frac{{y_{g} }}{y}\sim N(\mu_{g} ,\sigma_{g}^{2} )$, where the mean *μ*_*g*_ is the observed value of $2\sigma_{R}^{2} \frac{{y_{g} }}{y}$ from the dataset and the variance *σ*_*g*_^2^ is the observed value of $\left( {2\sigma_{R}^{2} \frac{{y_{g} }}{{y\sqrt {n_{g} } }}} \right)^{2} ,$ with *n*_*g*_ being the number of events (such as amenable deaths) in the *g*th income group. We then re-estimated the *C* by replicating the regression estimation 10,000 times to account for the uncertainty. The lower and upper limits of the 95 % confidence interval of the *C* were obtained as the 2.5 and 97.5 percentiles of the distribution of the simulated slopes. The median of the distribution of the slopes is equal to the *C* calculated from the observed dataset, determined by setting the observed values of the dependent variable in Eq. (2) as the expected values in the simulations. The property that the median of the distribution of the slopes is equal to the *C* results straight from the normal distribution assumption, since the median and the mean are equal by definition. This produces symmetrical confidence intervals around the observed *C*.

If there is a reason to assume larger errors (for example due to consistent miscoding of some variable), the variance *σ*_*g*_^2^ can be enlarged by reducing the factor of the denominator *n*_*g*_. This would indicate fewer events in an income group and thus assume more variation. Changing the size of the disturbance, however, would not change the median of the distributions (i.e., the *C*), but naturally it would enlarge the confidence intervals of the *C*.

The second model (denoted as MC rate) assumes that the age-adjusted rates follow normal distributions $y_{g} \sim N(y_{g} ,\frac{{y_{g}^{2} }}{{n_{g} }}),$, and both methods (1) and (2) can be used to estimate the confidence intervals.

In the next approach, we made more assumptions and developed the MC technique further to model the uncertainty in more detail. A requirement for the independence of the error terms is not needed because this method does not use errors estimated from a regression. It repeats the estimation of the *C* by allowing some variability in the health outcome (events) by income groups and also in the total number of events. Both methods (1) and (2) can be used to assess the *C*. We denote this technique as BIN. The variability is approximated from the observed data with the following assumptions and steps such as:

1. The observed *p*_*ig*_ (the population size), the denominator of the rate, is held fixed in the simulation. This is based on the assumption that the information on age and person-years in the registers is perfect.
2. The second assumption concerns the events that are treated as being random. The number of events is allowed to vary due to fact that there is some error in the coding of the events (such as causes of deaths). The *observed total* number of events in age group *i* is the sum over income groups ∑ _*g*=1_^*G*^ *d*_*ig*_ = *D*_*i*_.
3. The number of events is allowed to vary between income groups within the age group. This is permitted because the income information presumably does not exactly measure the person’s real wealth. It might not describe the real wealth level of a person since all assets are not recorded in the administrative registers. Income is obtained from multiple administrative sources and, in addition, may vary considerably even over a short period. Now, the number of events in group *ig* is simulated assuming to follow a binomial distribution *d*_*ig*_ ∼ *B*(*D*_*i*_, *ρ*_*ig*_). The denominator *D*_*i*_ is the same for all income groups within the same age group. Probabilities *ρ*_*ig*_ (with the constraints that ∑_*g*=1_^*G*^ *ρ*_*ig*_ = 1 and 0 ≤ *ρ*_*ig*_ ≤ 1) are estimated from the observed data $$ \rho_{ig} = {\raise0.7ex\hbox{${d_{ig} }$} \!\mathord{\left/ {\vphantom {{d_{ig} } {D_{i} }}}\right.\kern-0pt} \!\lower0.7ex\hbox{${D_{i} }$}}. $$
4. Simulation step (3) is repeated *N* times; thus, *N* is the number of simulated datasets.
5. Next, *N* sets of age-adjusted rates are calculated using the simulated number of events, the observed person-years at risk from the original dataset, and the weights from the original standard population.
6. Now, *N* values of the *C* are calculated using methods (1) or (2) from the simulated data yielding a distribution of the *C*. The 2.5 and 97.5 percentiles of this distribution comprise the 95 % confidence intervals for the *C*. Binomial distribution may not be symmetric, but with large *n* and not too extreme *ρ*_*ig*_, it is in practice quite often very symmetric. Thus, the median of the distribution of the slopes is likely very close to the *C* calculated from the observed data.

To test the robustness of the above-mentioned simulation techniques to estimate the confidence interval for the *C*, we performed more analyses using different assumptions. The fourth model (denoted POIS) is a simulation approach equivalent to BIN except the number of events in the step (3) follows a Poisson distribution *d*_*ig*_ ∼ *Pois*(*λ*_*ig*_) where the parameter *λ*_*ig*_ is the observed number of events in a group *ig*. Poisson distribution is asymmetrical when the mean is small. However, as the mean becomes large, the distribution becomes more and more symmetric and approaches normal distribution.

In the fifth simulation method (denoted MN), the total number of events within age group *D*_*i*_ is held fixed. The number of deaths is, however, allowed to vary between income groups within each age group. The number of events is simulated from a multinomial distribution with parameters *D*_*i*_ and *ρ*, and mean *E*{*X*_*ig*_} = *D*_*i*_ *ρ*_*ig*_ with the constraint that ∑ _*g*=1_^*G*^ *X*_*ig*_ = *D*_*i*_. The probabilities *ρ*_*i*_ = {*ρ*_*i*1_, …, *ρ*_*iG*_} (with constraints ∑ _*g*=1_^*G*^ *ρ*_*ig*_ = 1 and 0 < *ρ*_*ig*_ ≤ 1) are estimated from the observed data $$\rho_{ig} = {\raise0.7ex\hbox{${d_{ig} }$} \!\mathord{\left/ {\vphantom {{d_{ig} } {D_{i} }}}\right.\kern-0pt} \!\lower0.7ex\hbox{${D_{i} }$}}.$$

Table 1 presents all five methods and related modelling assumptions.

**Table 1:** Assumptions of the simulation methods

Method	Variable simulated	Modelling assumptions	Population assumptions	Estimation of the C
MC	$2\sigma_{R}^{2} \frac{{y_{g} }}{y}$	$2\sigma_{\text{R}}^{2} \frac{{y_{g} }}{y} \sim N\left( {\mu_{g} ,\sigma_{g}^{2} } \right)$	*μ*_*g*_ is the observed value of $2\sigma_{R}^{2} \frac{{y_{g} }}{y}$	The regression method (2)
			*σ*_*g*_^2^ is the observed value of $\left( {2\sigma_{g}^{2} \frac{{y_{g} }}{{y\sqrt {n_{g} } }}} \right)$	
			*n*_*g*_ is the number of events in the income group	
MC rate	*y*_*g*_	$y_{g} \sim N\left( {y_{g} ,\frac{{y_{g}^{2} }}{{n_{g} }}} \right)$	*y*_*g*_ is the observed rate of the event	The arithmetic method (1) or the regression method (2)
BIN	*d*_*ig*_	*d*_*ig*_ ∼ *B*(*D*_*i*_, *ρ*_*ig*_)	*D*_*t*_ is the observed number of events in age group i:∑ _*g*=1_^*G*^ *d*_*ig*_ = *D*_*i*_	Method (1) or (2)
		The population size in group *ig* is held fixed	*ρ*_*ig*_ = *d*_*ig*_/*D*_*i*_	
		The number of events is allowed to vary between income groups within the age group		
POIS	*d*_*ig*_	*d*_*ig*_ ∼ *Pois*(*λ*_*ig*_)	*λ*_*ig*_ is the observed number of events in group *ig*	Method (1) or (2)
MN	*d*_*ig*_	*d*_*ig*_ follows a multinomial distribution with parameters D, and *ρ* and mean *E*{*X*_*ig*_} = *D*_*i*_ *ρ*_*ig*_ with the constraint that ∑ _*g*=1_^*G*^ *X*_*ig*_ = *D*_*i*_	The probabilities $\rho_{{i}} = \left\{ {\rho_{{{{i}}1}} , \ldots ,\rho_{{ig}} } \right\}$ (with constraints ∑ _*g*=1_^*G*^ *ρ*_*ig*_ = 1 and 0 < ρ_ig_ ≤ 1) are estimated from the observed data: *ρ*_*ig*_ = *d*_*ig*_/*D*_*i*_	Method (1) or (2)
		The number of events within age group *D*_*i*_ is held fixed		
		The number of deaths is allowed to vary between income groups within each age group		

## Empirical example

As an empirical example, we used Finnish register data on deaths amenable to health care interventions. Monitoring socioeconomic inequalities in mortality amenable to health care interventions—which is used to measure health system performance based on certain premature deaths that should not occur if health care works effectively and is timely—provides useful information on changes in differentials in health service utilization and effectiveness (Schwarz and Pamuk 2008). These amenable deaths are an indication of potential weaknesses in health care that can then undergo more in-depth investigation (Nolte and McKee 2004).

Our dataset comprised all resident Finnish citizens aged 1–74 in 1996–2008. For this population, we received yearly information on deaths from an amenable cause including individual demographic and socioeconomic variables such as income, age, and gender. Due to data protection regulations, all variables were categorized. By means of unique identification codes, the information on mortality came from the cause-of-death register and the demographic variables came from the annual individual-level employment statistics database. Both registers are compiled and maintained by Statistics Finland. As an indicator of socioeconomic status to study equality, we had disposable family net income, adjusted for family size on the OECD equivalence scale (OECD 1982) and categorized into 20 income groups according to the Finnish income distribution, separately for each year. The income record applied was for the year before death. Age was grouped from 1 to 4 years and then in 5 year age bands.

The selection of causes of death considered amenable to health care focuses on conditions for which effective clinical interventions exist in people <75 years old and in this study was an adaptation of classifications by Page et al. (2006), Nolte and McKee (2008), and McCallum et al. (2013) (Table 2). Causes of death (as a main cause) were coded according to the 10th Revision of the International Classification of the Diseases (ICD).

**Table 2:** List of causes of death considered amenable to health care and corresponding ICD-10 codes

Place of intervention	Cause of death	Age	ICD-10
*Primary health care*			
Timing of intervention			
Primary prevention	Intestinal infections	1–14	A00–09
	Diphtheria, Tetanus, Poliomyelitis, and Varicella	1–74	A35–36, A80, B01
	Whooping cough	1–14	A37
	Measles	1–14	B05
	Rubella	1–74	B06
	Scarlatina	1–74	A38
	Meningococcus	1–74	A39
	Erysipelas	1–74	A46
	Legionellosis	1–74	A48.1
	Malaria	1–74	B50–54
	Streptococcal pharyngitis	1–74	J02.0
	Cellulitis	1–74	L03
Early detection and treatment	Tuberculosis	1–74	A15–19, B90
	Malignant neoplasm of colon and rectum	1–74	C18–21
	Melanoma of skin	1–74	C43
	Malignant neoplasm of skin	1–74	C44
	Malignant neoplasm of breast	1–74	C50
	Malignant neoplasm of cervix uteri	1–74	C53
	Malignant neoplasm of cervix uteri and body of uterus	1–44	C54–55
	Malignant neoplasm of bladder	1–74	C67
	Benign tumors	1–74	D10–36
	Hypertensive disease	1–74	I10–13.I15
	Cerebrovascular disease	1–74	I60–69
Improved treatment and medical care	Diseases of the thyroid	1–74	E00–07
	Diabetes mellitus	1–49	E10–14
	Epilepsy	1–74	G40–41
	All respiratory diseases (excl. pneumonia/influenza)	1–14	J00–09, J20–99
	Asthma	15–49	J45–46
	COPD	15–49	J40–44
*Specialized health care*	Septicemia	1–74	A40–41
	Malignant neoplasm of testis	1–74	C62
	Hodgkin’s disease	1–74	C81
	Leukemia	1–44	C91–95
	Rheumatic and other valvular heart disease	1–74	I01–09
	Influenza	1–74	J09–11
	Pneumonia	1–74	J12–18
	Peptic ulcer	1–74	K25–28
	Appendicitis	1–74	K35–38
	Abdominal hernia	1–74	K40–46
	Cholelithiasis and cholecystitis	1–74	K80–81
	Nephritis, nephrosis, and nephropathy	1–74	N00–09,N17–19,N25–27
	Obstructive uropathy and prostatic hyperplasia	1–74	N13,N20–21,N35, N40
	Maternal death	All	O00–99
	Congenital cardiovascular anomalies	1–74	Q20–28

When age-standardized values were required, we calculated annual amenable mortality rates (per 100,000 person-years) for 20 income groups in 1996–2008. The age- and income-specific rates, i.e., the number of amenable deaths as a proportion of person-years in the follow-up of the corresponding population, were directly age-standardized to the European standard population (Waterhouse et al. 1976).

We repeated the simulation approaches 10,000 times in our analyses; thus, *N* was 10,000, which we found to be a sufficient number of runs, since adding more runs did not change the lengths of the confidence intervals. The computer time with 10,000 repetitions for one simulation was negligible using a standard computer system for all methods. We used SAS (SAS Institute Inc., Cary, NC, USA) version 9.2 to analyze the data.

## Results

### Overview of data

In Finland in 1996, according to our definition, the total number of deaths considered amenable to health care interventions was 4087, of which 52 % occurred among men. The number of amenable deaths decreased evenly during the follow-up (*p* value for linear trend < 0.01), and by 2008, there were 3012 such cases (53 % among men). In 1996, the overall age-standardized rate per 100,000 person-years was 102 among men and 75 among women, and 60 and 50, respectively, in 2008. The average annual population (person-years) at risk was 4789,000 during the follow-up.

### Empirical findings

**Fig. 1:**
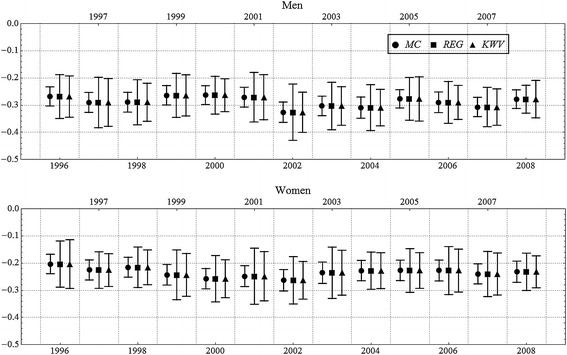
Concentration index and 95 % confidence intervals for amenable mortality using three different estimation methods

Figure 1 shows the concentration index and confidence intervals for mortality amenable to health care using three different techniques (MC, REG, and KWV*)* for each year 1996–2008 among men and women. The concentration index is estimated using data aggregated by 20 income groups. The middle of the vertical line is the *C* that is calculated from the observed data using the formula or the WLS method and is the same for all three techniques. The solid circle indicates the MC simulation technique. The ends of the vertical line represent the 2.5 and 97.5 percentiles of the Monte Carlo simulated distribution of the *C.* The second method (indicated with a square) is a basic regression method (REG) carried out applying procedures using a standard statistical software package. We also used the method proposed by Kakwani et al. (1997), which corrects for possible serial correlation (KWV). On average, the confidence intervals were over twice as wide with REG and KWV than with the simulation approach. The average of the lengths of the intervals was 0.16 among men and 0.17 among women with REG and 0.07 among men and 0.08 among women with MC. The correction for serial correlation did not have a notable effect on the length of the confidence interval (the average was 0.15 among both genders with KWV), though the intervals were generally narrower when this was taken into account.

All simulation techniques developed in this study yielded very similar results to MC in relation to the lengths of the confidence intervals of the *C* and thus showed that the results were consistent irrespective of the approach used and parametric assumptions (Table 3). The *C* in Table 3 is calculated from the observed data; the results were equivalent using the formula (1) or the regression model (2). Neither simulating events or rates instead of the outcome variable in formula (2), nor using binomial, multinomial, or Poisson distributions, nor keeping mortality fixed within the age groups, had any effect on the length of the confidence intervals. All methods, except the POIS method, resulted symmetrical confidence intervals around the *C* calculated from the observed data. The distribution of the simulated data in the POIS method was inherently skewed to the right due to the low mean values among the young age groups and the high-income groups, causing asymmetric confidence intervals.

**Table 3:** Sensitivity analysis for estimating 95 % confidence intervals for the concentration index (*C*) for amenable mortality

	1996			2000			2004			2008		
Method	C^1^	95 % CI	1(C)^2^	C^1^	95 % CI	1(C)^2^	C^1^	95 % CI	1(C)^2^	C^1^	95 % CI	1(C)^2^
*Men*												
MC	−0.27	−0.30 to −0.23	0.07	−0.26	−0.30 to −0.23	0.07	−0.31	−0.35 to −0.27	0.08	−0.28	−0.31 to −0.24	0.07
MC rate	−0.27	−0.30 to −0.23	0.07	−0.26	−0.30 to −0.23	0.07	−0.31	−0.35 to −0.27	0.08	−0.28	−0.31 to −0.24	0.07
BIN	−0.27	−0.30 to −0.24	0.07	−0.26	−0.30 to −0.23	0.07	−0.31	−0.34 to −0.27	0.07	−0.28	−0.31 to −0.24	0.07
POIS	−0.27	−0.32 to −0.24	0.08	−0.26	−0.30 to −0.24	0.05	−0.31	−0.36 to −0.28	0.08	−0.28	−0.33 to −0.25	0.08
MN	−0.27	−0.30 to −0.24	0.07	−0.26	−0.30 to −0.23	0.07	−0.31	−0.34 to −0.27	0.07	−0.28	−0.31 to −0.24	0.07
*Women*												
MC	−0.20	−0.24 to −0.17	0.07	−0.26	−0.29 to −0.22	0.07	−0.23	−0.26 to −0.19	0.07	−0.23	−0.27 to −0.19	0.08
MC rate	−0.20	−0.24 to −0.17	0.07	−0.26	−0.29 to −0.22	0.07	−0.23	−0.26 to −0.19	0.07	−0.23	−0.27 to −0.19	0.08
BIN	−0.20	−0.24 to −0.17	0.07	−0.26	−0.29 to −0.23	0.07	−0.23	−0.26 to −0.19	0.07	−0.23	−0.27 to −0.20	0.07
POIS	−0.20	−0.26 to −0.19	0.08	−0.26	−0.33 to −0.23	0.10	−0.23	−0.30 to −0.20	0.10	−0.23	−0.30 to −0.21	0.09
MN	−0.20	−0.24 to −0.17	0.07	−0.26	−0.30 to −0.23	0.07	−0.23	−0.26 to −0.19	0.07	−0.23	−0.27 to −0.20	0.08

^1^The concentration index (*C*) is obtained from observed data

^2^Length of the confidence interval

Additionally, we compared the results of our MC-rate method with empirical results drawn from the data. This was done by calculating the variance from the empirical data by looking at the rates (*y*) in each SEG across certain years (*j*) and calculating the variance of the rates in each SEG [i.e., var(*y*_*gj*_)]. We used three different sets of years such as: 1) two preceding years and the study year, 2) two preceding years, the study year and two next years, and 3) three preceding years, the study year and three next years. The lengths of the confidence intervals of the *C* were equivalent with the results shown in Table 3, giving an empirical justification to our introduced methods.

## Discussion

This paper concerns statistical inference on the concentration index calculated from aggregated population register data. The *C* is a tool, which can be used to measure socioeconomic equality in health and health care. We present several approaches to estimate the uncertainty of the *C* in a novel way which take into account the nature of the population data. This fills a gap in the literature, as previous studies assessing equality with the *C* have mainly used sample or survey data and have not addressed the use of register data. The confidence intervals for estimates using sample data do not account for sources of uncertainty other than sampling error, including missing and incomplete data and other data errors, bias resulting from non-response, and poor data collection. Evaluation of the uncertainty of the estimates ensures that comparisons of equality at different levels (between hospitals, areas, countries, in time) are meaningful. In this study, we focus on register data, discuss what kind of uncertainties exist when using them, and how to model them when calculating the *C*. However, several factors can affect the uncertainty complicating this calculation. Even within Europe, countries have register data of different quality (Kunst 1997). Countries also differ regarding data collection, practices, and recording information on death certificates. Thus, the uncertainty of the inequality estimates should be evaluated on a case-by-case basis.

For all data types (register or survey), the *C* is conveniently calculated using the regression method or the original arithmetic formula. Moreover, the application of regression techniques for point estimation does not require distributional assumptions. On the other hand, making any statistical inferences regarding the uncertainty of the *C*—using the standard error of the regression slope as an estimate of the standard error of the *C*—the distributional assumptions need to hold. As Wagstaff and van Doorslaer (2000) have suggested, serial correlation in the errors potentially causes biased standard errors. Therefore, testing for serial correlation is recommended when making statistical inferences using the regression method. Our empirical illustrations suggest, however, that the difference between taking serial correlation into account (KWV) and failing to do so (REG) when estimating the uncertainty of the *C* is small, which is in line with earlier studies (Kakwani et al. 1997; Chen and Roy 2009). According to our findings, both the convenient regression method (REG) and the KWV method generally overestimate the standard errors for the *C* in the case of population register data. In some cases, however, the regression method might underestimate the error, for example if the observations from the dataset fit a regression line accurately. A recent paper by Chen et al. (2012) shows using fully simulated individual-level (sampled) data that the level of uncertainty of the *C* may be dependent on the value of the *C* and the regression method underestimates the standard error when the *C* is >0.2. Yet, the difference between the estimates obtained from a correction for serial correlation and from the regression method is small when the *C* is <0.2. The comparison of these divergent results this study, and Chen’s is not, however, straightforward since the simulation settings and underlying assumptions are very different. Thus, depending on the setting and the form of the distribution of the studied event, the linear regression method may either under- or overestimate the true standard error of the *C*.

The standard error of the regression slope describes the variability in the estimate around the true slope parameter. It takes into account the error occurring in fitting a regression line for the observed data. Nevertheless, does the uncertainty of the regression slope represent the uncertainty of the concentration index and corresponding concentration curve? An alternative way of computing probability intervals is through simulation that overcomes the problems of serial correlation or lack of linearity. For example, bootstrap and jackknife techniques have been shown to be superior to asymptotic intervals both theoretically and in a variety of applications using inequality measures (Mills and Zandvakili 1997; Sergeant and Firth 2006). Modarres and Gastwirth (2006) show using individual-level data that using the regression method to estimate the standard error of the Gini coefficient yields standard errors that are too large due to serial correlation, and they recommend using jackknife and bootstrapping methods. Our results show that the regression method estimates the uncertainty in the *C* too conservatively for aggregated register data, since this method does not take into account the extensive dataset underlying the points of the regression. In addition, our simulation methods to estimate confidence intervals for the *C* have the advantage that the serial correlation between the rank and the outcome variable do not bias results since we do not estimate standard errors using the regression method. The simulation of point estimates avoids this problem since the possible correlation structure between the ranks (of income group) and outcomes (mortality) remains approximately the same in the replicates as in the original data. The similar results obtained using slightly different assumptions support the interpretation that serial correlation between the rank and outcome is not an issue in our simulations. Our results also showed that the point estimates and confidence intervals were systematically equivalent using regression or formula methods. Since, by definition, serial correlation is not present using the original formula method, this result means that it is not an issue in our simulations. Furthermore, using the regression method, the error is evaluated mainly based on the size of the error terms in the Eq. (2). Thus, if the observed relationship between the dependent variable and the rank variable is not linear across the socioeconomic groups, the error will be estimated to be larger than if the relationship were linear. Mills and Zandvakili (1997) also note that inequality indices are nonlinear functions of a random variable (such as income), and so do not readily lend themselves to standard statistical techniques. In the real world, the uncertainty of the data is, however, caused by other factors and is neither due to the lack of a linear relationship between the health care variable and the rank nor due to outliers in a regression sense. Consequently, in relation to register data, using the regression method to calculate standard errors does not factor in any inaccuracy in the observed data. However, in survey studies, regression methods allow taking complex sampling designs into account (O’Donnell et al. 2008). Simulation techniques have the disadvantage that they may be mathematically complex or require a considerable amount of numerical computation. Thus, applications which are easily utilized and are suitable for different scenarios are of major importance.

We obtained consistent results from our sensitivity analyses corroborating the suitability of the introduced methods, suggesting that the distributional assumptions used have a rather small effect on the lengths of the intervals. The method simulating the outcome variable of Eq. (2) (MC); the method simulating adjusted rates (MC rate); and the methods BIN, MN, and POIS simulating the crude observed data (i.e., unadjusted events) provided equivalent confidence intervals. Although the method using the Poisson distribution introduced confidence intervals whose lengths differed between years, the differences were negligible. In addition, the POIS method yielded slightly asymmetrical confidence intervals. For the MN method, we studied the effect of keeping the total number of events fixed in each age group. In our example, this had a minor impact on the confidence intervals. This is due to the fact that the number of deaths from amenable causes is relatively low. The assumption of a fixed number of events would be reasonable when studying total mortality or other similar events using reliable data without misclassification problems. On the other hand, allowing the number of deaths in each age group to vary may be reasonable in situations where only some of the events are being studied and the misclassification or miscoding may cause some uncertainty, with false-negative and false-positive cases. However, some random number generator procedures in statistical programs do not allow zero probabilities. Thus, when studying rare events, it might be more suitable to use a random number generator allowing zero probabilities. In addition, we suggest that researchers should test different distributions in their own studies to find the best method for the specific situation. Despite the fact that we found a more equitable distribution of amenable deaths between income groups among women than men, the findings concerning estimates of the confidence intervals were consistent between genders.

Our empirical example of comprehensive Finnish register data on amenable mortality covers the years from 1996 to 2008. Although amenable mortality faces some criticism as a straightforward indicator of the quality of health care, inequalities in amenable mortality illustrate a broad picture of the state of equality in health care as the deaths also cover those groups who do not use or do not have proper access to health services (Nolte and McKee 2004). In this study, we were not able to take into account the possible effect of differences in the prevalence and incidence of some diseases between socioeconomic groups on disparities in amenable mortality. Inequalities in the use of and access to health services may, however, affect the incidence of subsequent disease.

In Finland, register data are generally of high quality (Gissler and Haukka 2004). It has been shown for several Finnish administrative registers that all or nearly all events are included (Gissler and Shelley 2002; Sund 2012). Regardless of the quality of the data, some variation may occur in the coverage and validity of some variables (Gissler and Haukka 2004). Lahti and Penttilä (2001) studied the validity of death certificates in Finland and its effects on mortality statistics. While their findings confirmed that the data are on the whole of good quality, variation exists in the underreporting of some causes of deaths as the underlying cause of death. In addition, about 7 % of certificates are not completed as instructed. Manderbacka et al. (2013) found that a large part of the decline in pneumonia mortality from 2000 to 2008 was due to changes in coding practices. Also, they reported large regional variation in coding practices. One study also suggests that the official classification of maternal deaths in Finland is rather arbitrary and allows a lot of variation in the definition of a maternal death (Gissler et al. 1997).

## Conclusions

In order to achieve an equitable distribution of health and health services between socioeconomic groups, we need a better knowledge of inequalities in health and the use of health services. This requires more precise measures of equality as well as detailed information on indicators for health and health care use. The approaches we introduce in this study for the concentration index can also be adapted to other measures of equality in health, such as the relative index of inequality and the slope index of inequality. With these techniques, it is possible to evaluate comparable confidence intervals for the population estimate on a case-by-case basis. Although the assessment of uncertainty is demanding, our approaches provide substantive better approximation than the regression method or the KWV method. Consequently, we recommend using our simulation technique to estimate the confidence intervals for the *C* when assessing equality using grouped register data.
